# Transition from Laser to Intravitreal Injections for Diabetic Retinopathy: Hospital Utilization and Costs from an Extended Healthcare Perspective

**DOI:** 10.3390/ijerph191912603

**Published:** 2022-10-02

**Authors:** Silvia Nanjala Walekhwa Hertzberg, Øystein K. Jørstad, Beáta Éva Petrovski, Ragnheidur Bragadottir, Leif Arthur Steffensen, Morten Carstens Moe, Emily A. Burger, Goran Petrovski

**Affiliations:** 1 Center for Eye Research, Department of Ophthalmology, Institute for Clinical Medicine, University of Oslo, 0450 Oslo, Norway; 2 Department of Ophthalmology, Oslo University Hospital, 0450 Oslo, Norway; 3 Human Resources, Oslo University Hospital, 0424 Oslo, Norway; 4 Department of Health Management and Health Economics, University of Oslo, 0317 Oslo, Norway; 5 Center for Health Decision Science, Harvard T. H. Chan School of Public Health, Boston, MA 02115, USA; 6 Department of Ophthalmology, University of Split School of Medicine and University Hospital Centre, 21000 Split, Croatia

**Keywords:** diabetic retinopathy, intravitreal injections, laser treatment, vitrectomy, healthcare costs

## Abstract

Purpose: To describe the trends in hospital utilization and economic outcomes associated with the transition from laser to intravitreal injection (IVI) therapy for diabetic retinopathy (DR) at Oslo University Hospital (OUH), which provides the largest retina service in Norway. Methods: This descriptive study analyzed hospital administrative data and determined the average utilization and treatment proportions of laser therapy, IVIs and vitrectomy for each patient per year. The Chi-square test was used to compare resource use between treatment groups. From an extended healthcare perspective, the annual cost per patient was calculated using Norwegian tariff data from 2020 and the National Medication Price Registry for patients seen between 2010 and 2018. Bootstrapping was performed to generate 95% confidence intervals for the cost per patient per year. Results: Among the 1838 (41% female) patients treated for DR between 2005 and 2018, OUH provided on average 1.09 laser treatments per DR patient and 0.54 vitrectomies per DR patient in 2005, whose utilization declined to 0.54 and 0.05 treatments per DR patient, respectively, by 2018. Laser treatments declined from 64% to 10%, while vitrectomies declined from 32% to 1%. In contrast, IVI treatments increased from 4.5% to 89% of the total share, representing an average increase, from 0.08 injections per patient in 2005 to 4.73 injections per patient in 2018. Both the increasing number of DR patients and the shift in the type of treatment increased the economic costs of treating DR from a total of EUR 0.605 million (EUR 2935 per patient) in 2010 to EUR 2.240 million (EUR 3665 per patient) in 2018, with IVIs contributing considerably to these costs. Conclusions: Despite the decline in the use of vitrectomies, the transition from laser to IVI therapy for DR increased the healthcare resource utilization and economic costs of its treatment over the observed time. A main cost driver was the need for long-term IVIs, in addition to the drug cost itself. Trade-offs can be achieved through effective alternative IVI delivery or appropriate drug choice that balances patient needs with the economic burden of treating DR.

## 1. Introduction

Diabetic retinopathy (DR) is a complication of diabetes mellitus (DM) and primarily affects the small retinal vessels. Globally, DR is a major cause of blindness, especially among working-age individuals [1]. The burden of DR is likely to escalate in the future, accompanied by an increasing prevalence of DM. Studies have projected that by the year 2045, 628.6 million people will suffer from DM, of whom one third are expected to develop DR [2,3].

Early diagnosis and treatment of DR are critical, as treatment can halt further progression and prevent blindness. DR is classified according to severity levels: (1) no apparent retinopathy, (2) mild, moderate, or severe non-proliferate diabetic retinopathy (NPDR), and (3) proliferative diabetic retinopathy (PDR) [4]. Notoriously, DR often remains asymptomatic until development of PDR or diabetic macular edema (DME) [2].

Historically, the treatment of DR has predominantly involved laser photocoagulation (2–3-day sessions). Laser therapy is still considered the main treatment for PDR; however, it has poor potential to improve visual acuity (VA) [5]. On the other hand, intravitreal injection (IVI) of antiangiogenic biologics (e.g., bevacizumab, ranibizumab and aflibercept) or corticosteroids (e.g., triamcinolone acetonide, dexamethasone and fluocinolone) has significantly transformed the management of DR, improving the visual prognosis of DME compared to the conventional laser therapy [6]. The better visual outcomes associated with IVI therapies have been attributed to the immediate release of the drug to the targeted tissue compared to laser therapy [7,8,9,10,11]. 

Late stages of DR may require vitreoretinal surgery to improve the oxygenation of the retina and remove fibrous membranes and opacities in the vitreous body, as well as in the posterior hyaloid [12,13]. Even though vitreoretinal surgery is considered highly effective and safe in treating severe cases, patients may experience side effects, e.g., inflammation, blurred vision and post-vitrectomy cataracts, resulting in unfavorable visual outcomes [14].

The DME and DR severity levels of the individual patient are the main determinants for the choice of treatment [15]. A study on the treatment preferences among DR patients found that visual function influenced the treatment choice [16]. This finding was supported by another study, in which patients turned down a less frequent laser treatment and instead preferred a more frequent IVI treatment, as the latter better improved the visual prognosis [17]. Given the effectiveness of IVIs, there is currently a preference for IVIs in treating DR in even earlier than severe levels or in treating DME. However, this poses challenges in providing sufficient patient care as well as challenges in resource availability [18,19,20,21]. As IVIs require frequent retreatment—e.g., on a monthly basis (or using the treat-and-extend protocol at 4-, 8- or 12-week intervals)—in regard to the drug used, the costs and patient time commitments may vary greatly.

In line with the strong evidence, Norwegian ophthalmologists have gradually adapted IVIs for DR therapy, largely replacing conventional laser therapy for DME. Still, little is known about the state of DR therapy in Norway over longer periods of time. Specifically, the need for prolonged IVIs may increase the burden of managing DR compared to potentially permanent laser therapy. 

In this study, we aim to describe the trends in hospital utilization and economic outcomes associated with the transition from laser to IVI therapy for DR during the period between 2005 and 2018, including trends in the use of vitreoretinal surgery in the same time period at the Department of Ophthalmology, Oslo University Hospital (OUH).

## 2. Methods

### 2.1. Study Design and Patient Population

This is a retrospective, registry-based study, which descriptively quantifies the resource use and economic costs of managing DR over time in a large retina service setting. The study took place at the Department of Ophthalmology, OUH, which provides the largest retina service in Norway. The use of registry data was approved by the institutional data protection officer in accordance with the General Data Protection Regulation (20/08877). All patients diagnosed with and treated for DR were included irrespective of the type of diabetes and sex, and the average age per year per treatment was recorded.

Hospital statistics were searched for episodes of care that included the Nordic Medico-Statistical Committee’s Classification of Surgical Procedures (NCSP) codes CKC12: Transpupillary laser treatment of the retina, CKD05: IVI of a drug, or CKD65: Pars plana vitrectomy, in connection with the International Classification of Diseases (ICD-10) diagnosis H36.0: Diabetic retinopathy or E10.3 and E11.3 for type 1 and type 2 diabetes, respectively. The same ICD-10 code was used for PDR and DME, thus, we could not separate treatments for these two conditions. For each episode, we registered the following parameters: patient-specific identification number, sex, the Anatomical Therapeutic Chemical (ATC) classification system drug code, and the NCSP code ZXA 00, ZXA 05, or ZXA 10: Right, Left, or Bilateral procedure. We counted a bilateral procedure or the use of two different drugs as two separate procedures. Initially, ATC codes for bevacizumab and ranibizumab were not routinely registered, and we could only provide an overview of anti-VEGF drugs from 2010 to 2018. We also included the use of the dexamethasone implant Ozurdex (Allergan, Dublin, Ireland).

Although the ICD-10 diagnosis H36.0 includes both DR and DME, these patients face differing treatment protocols. Panretinal laser photocoagulation (PRP) was generally the mainstay of treatment for PDR throughout the study period, but anti-VEGF drugs were increasingly used as complementary treatments. On the other hand, anti-VEGF drugs almost fully replaced laser therapy for DME during the same time period. With regard to the drug of choice, bevacizumab was generally used as the first-line treatment for DME in the beginning of the anti-VEGF era, with ranibizumab as the second-line treatment *pro re nata*. Aflibercept became commercially available in 2013 and was initially used as the second-line treatment in treatment-resistant cases. In 2015, on the basis of DRCR.net Protocol T, aflibercept became the first-line treatment in DME cases presenting with decimal visual acuity of <0.4 [22]. Meanwhile, the dexamethasone intravitreal implant became commercially available in 2010 and has since been used as an alternative to anti-VEGF drugs *pro re nata*. 

OUH serves as the local retina service for approximately one million people living in the city of Oslo and its nearby municipalities. Moreover, it is the regional vitreoretinal surgery service for about three million people living in Norway’s South-Eastern health region. Although this region has several other eye centers that perform laser and IVI treatment for diabetic eye diseases, vitrectomy only takes place at OUH. Consequently, the data generally represent laser and IVI treatment of our local patients, while vitrectomy data are for both local and regional patients. 

The majority of local patients were referred to OUH by private ophthalmologists, typically following DR screening or because of symptomatic DME. Regional patients were generally referred from local eye departments, which performed laser and IVI treatment but not vitreoretinal surgery. Choice of treatment at OUH (i.e., laser, IVI or vitreoretinal surgery) was at the ophthalmologist’s discretion, changing over time in line with the best available evidence.

### 2.2. Costs

In line with the national guidelines [23], an extended healthcare perspective (e.g., direct healthcare costs and two-way transportation costs, as well as time spent by the patient to travel to the care facility and receive treatment) was used to calculate the annual treatment costs between 2010 and 2018 for each patient receiving either laser or IVI therapy. Calculation of the treatment costs between 2005 and 2009 was not possible due to irregular code registration of the IVI drugs. The initial patient diagnosis costs were excluded, as these were incurred by patients irrespective of the treatment type. To estimate costs, we included all relevant expenses related to each treatment, including optical coherence tomography (OCT) costs, outpatient hospital visit costs, patient co-payment, two-way patient travel costs, patient time to receive each treatment, and intraocular pressure measurement costs (in particular, after IVI of dexamethasone implants).

For all treatments, laser and vitreoretinal surgery, the diagnostic related group (DRG) weight for a respective treatment was multiplied with the unit cost to estimate the cost of treatment [24] (Table 1). The costs for laser and surgical equipment were assumed to be included within the DRG weight [25]. The cost for IVI was based on recommendations by the Norwegian Medicines Agency (2020). Considering that the drugs may vary in their cost, and so may the cost of vial compounding at the hospital pharmacy (NoMA drug cost [26]), the injection drug costs were estimated individually as the costs per drug agent. For example, one bevacizumab vial is generally divided into 40 single IVI doses, whereas ranibizumab and aflibercept are divided into 2 and 2.5 IVI doses, respectively. Therefore, we divided the cost of the drug by the number of individual doses and added the cost for the vial. As dexamethasone drug implants are individually packed, no vial cost was attributed; however, this treatment requires an extra visit for measuring the intraocular pressure, which also incurs a cost in itself. 

For each patient, the number of visits for a given IVI treatment type (bevacizumab, ranibizumab, aflibercept or dexamethasone) was calculated and added to the total treatment cost for each year. The patient time required for each treatment at a given visit—the average time for laser therapy, IVI or vitreoretinal surgery, which was estimated to be 120 min, 90 min, and 150 min, respectively—was also added, including the estimated transportation time to and from our department. For each visit, we further added the transport costs for a two-way journey. The registry data could not indicate specific adverse events due to treatment. Therefore, these costs were not specifically included in the analysis. We applied the tariff and unit costs from 2020; therefore, the total treatment costs per year between 2010 and 2018 are expressed in 2020 Norwegian Kroner (NOK) and converted to Euros as per the 20 February 2020 exchange rate (NOK 1 = EUR 0.1) (Norges Bank).

### 2.3. Analyses and Outcomes

To explore the trends in resource use during the study period, we present the data descriptively by calculating the number of patients in each year, the number of each treatment type offered and the average number of laser therapies, IVIs and vitreoretinal surgeries per year between 2005 and 2018. To identify changes with the introduction of IVI therapy, we calculated the treatment percentage share for all three treatments (laser therapies, IVI and vitreoretinal surgeries) performed each year. The distribution and average differences between groups were tested using Chi-square (χ^2^) and multivariate tests, respectively, with a significance level of *p* < 0.05. In order to differentiate new patients from patients that may be continuing previous treatment protocols, we estimated the number of treatment-initiating patients per year as the number of new indexes per year. The difference between the total number of patients and the number of new patients in the year was considered to be the number of continuing patients for that year. Consequently, using the estimated continuing patients for each year in comparison to the previous year, we were able to establish patients either discharged or untreated in consecutive year(s). We estimated the average treatment cost per year per patient and performed bootstrapping to estimate the 95% confidence intervals. We explored the contribution of each drug used for IVI and estimated the cost associated to the drug. All statistical analyses were performed in STATA 16 [28] and Microsoft 365 (Excel).

## 3. Results

### 3.1. Hospital Utilization

**Figure 1:**
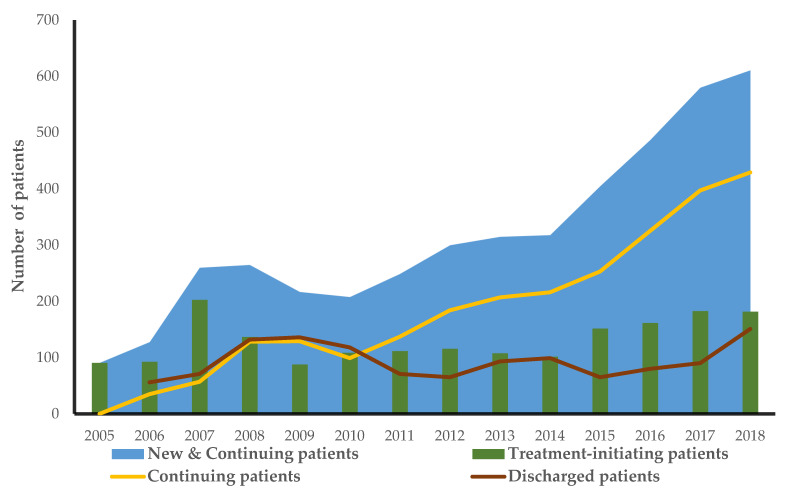
The annual number of new patients and those being treated the previous year, discharged patients, and patients in total.

The analysis included 1838 patients (41% females) diagnosed with and treated for DR between 2005 and 2018, increasing from 91 patients in 2005 to 611 in 2018 (Table 2). The patients’ average age ranged from 49 to 63 years, with the age of IVI patients having a varying upwards trend in the successive years. During the entire period, there were 5019 laser procedures with an average (±standard error) of 2.2 (±0.04) treatments per year per patient, 11969 IVIs with an average of 4.7 (±0.07) injections per year per patient, and 670 vitrectomies with an average of 1.2 (±0.02) surgeries per patient (Table 2). However, this resource usage was not constant over the study period. For example, the number of patients treated each year for DR increased from 91 (2005) to 611 (2018); the total laser treatment usage decreased from 64% in 2005 to 10% in 2018, while the average laser treatment usage declined from 1.09 (±0.14) per patient to 0.54 (±0.05) per patient in the same period. In contrast, total IVI treatment usage increased over time from 4.5% in 2005 to 89% in 2018, as the average IVI treatment usage increased from 0.08 (±0.03) in 2005 to 4.73 (±0.14) per patient in 2018. Finally, vitreoretinal surgery usage decreased from 32% in 2005 to 1% in 2018, as the average number of vitreoretinal surgeries performed declined from 0.54 (±0.06) in 2005 to 0.05 (±0.01) per patient in 2018 (Table 2). There were no statistical differences between sexes for any of the procedures, but there was a significant difference in the average number of treatments per year between the treatment groups (F statistic 386, 3-4430 degrees of freedom and *p*-value 0.0000). There were more new patients registered in 2007 followed by a decline until 2014, when we observed a steadily increasing pattern in the incidence of DR onwards. In the first years of the study, almost half of the patients were discontinued from their treatment regimen. From 2011, the gap between those retained on treatment (continuing patients) and those discontinued increased rapidly, with only a quarter of the patients in 2018 being discontinued accordingly (Figure 1).

**Figure 2:**
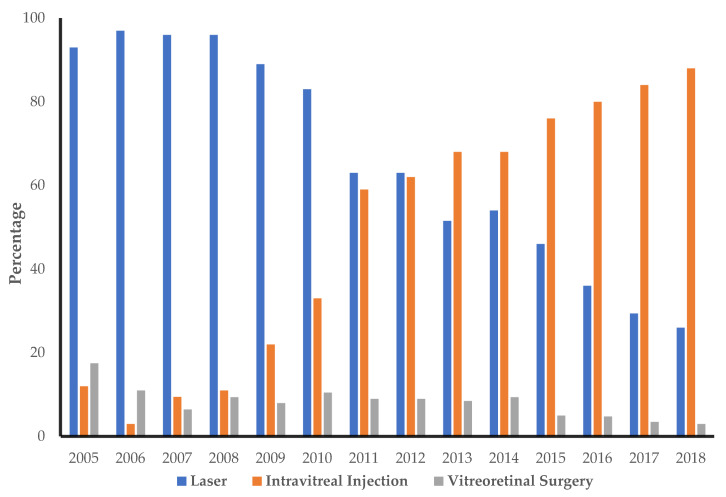
Average percentage of treatments for diabetic retinopathy in patients receiving laser, intravitreal injection and/or vitreoretinal surgery.

**Figure A1:**
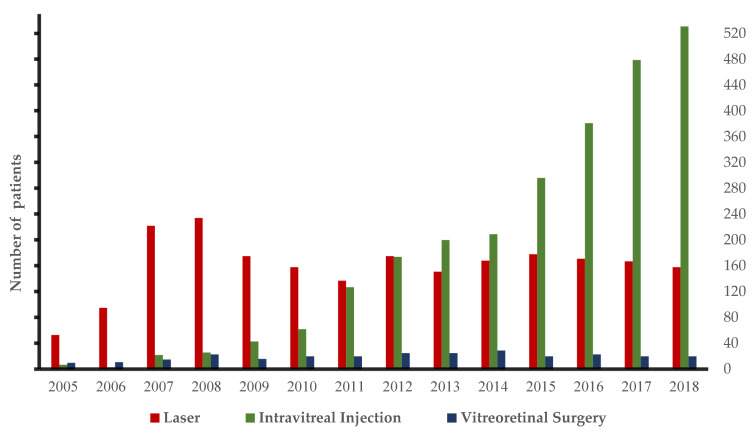
Number of treated patients per year receiving intravitreal injection, laser or vitreoretinal surgery (2005–2018).

**Figure A2:**
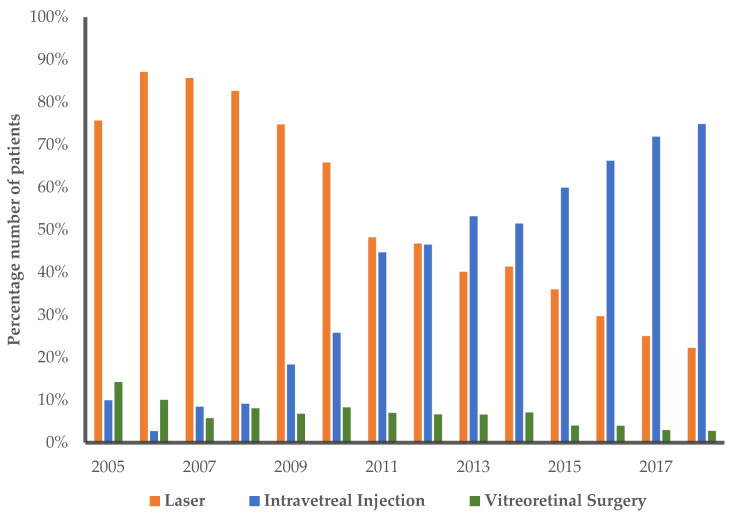
Percentage of the number of patients with diabetic retinopathy being treated with the different treatment modalities per year.

**Figure A3:**
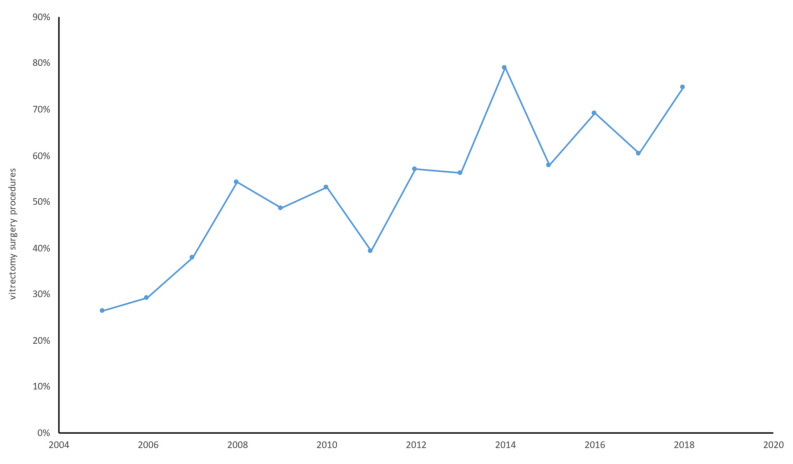
Vitreoretinal surgery procedures performed on patients who did not receive any other treatment for diabetic retinopathy at Oslo University Hospital. Among those who received vitreoretinal surgery, there was an increasing trend for patients who were treated with surgery alone; in this patient group, however, the hospital service area was wider than for those receiving intravitreal injections and laser only.

As a share of the total DR treatments at OUH, the proportion of laser treatments declined, and the proportion of IVIs increased over the 13 years of observation. As with laser treatment, vitreoretinal surgery showed a decreasing trend over time, with 3% of patients receiving vitreoretinal surgery in 2018 compared to 14% in 2005 (Figure A1 and Figure A2). Fewer vitreoretinal surgery treatments were performed in the recent years compared to the years prior to 2010, with a number of patients being previously treated by laser and/or IVIs undergoing fewer vitreoretinal surgeries (Figure 2 and Figure A3). The same treatment patterns over time were observed according to the sex (results not shown).

### 3.2. Treatment Costs

**Figure 3:**
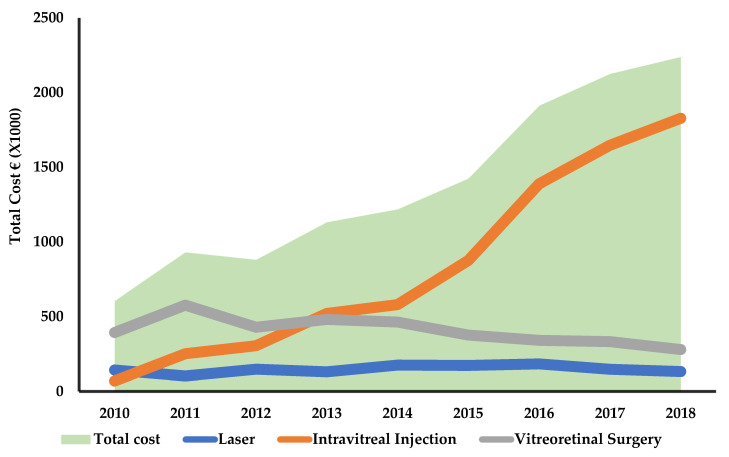
Annual total healthcare cost of treating diabetic retinopathy per modality and total treatment costs.

The total cost per year increased from EUR 0.605 to EUR 2.240 million, and the total cost per patient per year increased from EUR 2906 to EUR 3665 in the same time period (2010–2018). The total cost per patient per year, however, did not differ much in each successive year (Table 3 and Figure 3). In the laser group, the average cost per patient remained almost unchanged throughout the observation period, ranging from EUR 899 in 2010 to EUR 836 in 2018. In contrast, in the IVI group, the average cost per patient increased more than three-fold in less than 10 years, from EUR 1116 (2010) to EUR 3442 (2018). Meanwhile, the average cost of vitreoretinal surgery remained nearly the same throughout the study period, from EUR 10,348 in 2005 to EUR 10,357 in 2018 (Table 3).

In the laser treatment group, the treatment use declined, keeping the total cost of this treatment modality low. The cost of vitreoretinal surgery also declined since fewer surgeries were performed over the study period. IVIs caused a steep rise in cost from 2014 onwards in particular, which again increased the total cost of DR treatment (Figure 3). 

**Figure 4:**
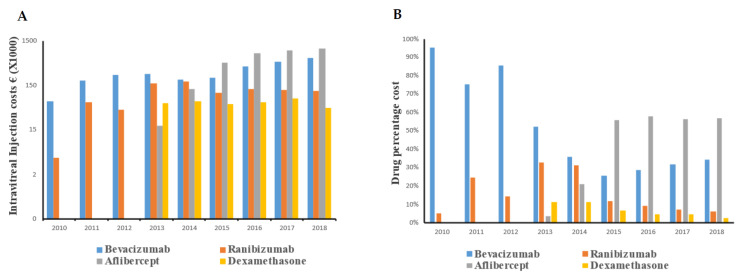
Total costs per year of each intravitreal drug (**A**) and percentage of the total cost per year for each of those drugs (**B**).

Corresponding to a steady increase in IVIs during the study period, the calculated drug cost expenses also increased. Initially, bevacizumab represented the main cost but was gradually replaced by aflibercept (Figure 4). Ranibizumab and dexamethasone were used less frequently over time, yielding lower costs (Figure 4). 

## 4. Discussion

This study focused on the transition from laser to IVI therapy for DR and aimed to estimate its costs from an extended healthcare perspective. As could be expected, we found a decline in the use of laser and the cost associated with such treatment over the 13-year study period. Vitreoretinal surgery showed similar results. Conversely, IVIs increased over the same time period, in particular from 2013 onwards, when all included IVI drugs became available in Norway, resulting in escalating IVI expenditures in the treatment of DR.

Our registry data could not directly measure the visual outcomes of treatment, but we observed a negative correlation between the transition from laser to IVI and vitreoretinal surgery. The percentage of patients receiving vitreoretinal surgery decreased almost five-fold from 2005 to 2018, indicating a declining trend in the development of severe DR demanding vitreoretinal surgery after the introduction of IVIs. This is in line with a study by Gross et al., which found fewer vitrectomies being performed in patients with PDR being treated with ranibizumab compared to laser [29]. In addition, vitreous hemorrhage is now commonly treated with IVIs, even though the visual recovery appears to be slower than after vitrectomy [30]. Accordingly, the total cost of vitreoretinal surgery decreased over time in our study.

Like other studies have found, the cost of treating DR gradually increased, nearly quadrupling the total expenditure (from EUR 0.605 million to EUR 2.240 million) in less than 10 years (2010–2018), mainly because of IVIs [31,32,33]. Our patients received approximately five IVIs per year; those who did not respond to the first-line bevacizumab treatment or had a decimal visual acuity of <0.4 were switched to aflibercept [34]. Taken together, this intensified the treatment cost of DR through both cost- and visit-associated resource increases.

Our study only considered an extended healthcare perspective, and it is essential to note that any given study can highly impact the cost results of the analysis [35]. Therefore, the costs would have been enormous if our study considered a societal perspective as well. For instance, in a study from Germany that analyzed DR cost from two perspectives (payer’s and societal), the total cost was 57% higher when the societal perspective was included, compared to the payer’s perspective [36]. 

In general, laser equipment is readily available in retina clinics, and from an extended healthcare perspective, laser treatment only involves costs for the visits, resource use, and relevant patient time. In this study, patients treated with laser had a total treatment usage of 10% in 2018, down from 64% in 2005. The decrease in laser procedures is assumed to be a result of better visual outcomes obtained with IVI, for DME in particular [6,37,38,39].

IVI of anti-VEGFs and corticosteroids for DR has largely replaced laser therapy as the standard of care. Still, more patients receive IVI over time, and our study also shows that IVI generally continues for several years. In this way, the contemporary preference for IVI casts a burden on both patients and healthcare institutions; the increasing shortage of ophthalmologists adds yet another burden [40,41]. DR patients have been known to endure an already existing healthcare economic burden due to their DM state. The need for repetitive IVI may further affect the patients’ quality of life and treatment compliance [33,42,43,44,45]. In the case of frequent IVI use, the latter has been associated with greater outcomes in saving visual loss. Even though some studies have suggested decreasing the frequencies of IVIs in this patient group, frequent use may be beneficial, depending on the drug administered. For instance, the dexamethasone implant has prolonged efficacy (an average of 3 injections per year), though it may require further visits to manage intraocular pressure-related issues [10,15,33,46,47,48,49]. 

Further recommendations have been made to reduce the burden of IVIs, such as the treat-and-extend algorithm and same-day bilateral injections, though developments are still ongoing. For instance, bilateral injections may reduce visit frequency, but lack clear evidence about possible adverse events associated with their implementation (such as endophthalmitis and intraocular inflammation) [50,51,52]. Among the patients with DME, findings in a few studies have indicated similar or non-significantly improved visual acuity for the treat-and-extend algorithm compared to monthly dosing [53,54] Such a trade-off needs further research in different hospital settings to estimate the outcome benefit of this practice in patients with DR. Introduction of new biologics, such as faricimab, may also reduce the treatment burden, as it can be injected with even longer intervals [55,56].

Our study shows an accumulation of DR patients receiving long-term IVI treatment. Consequently, the average age increased over time. On the other hand, patients receiving laser or vitreoretinal surgery had similar or even lower age over time, which indicates that patients who are treated for PDR are getting younger. Additionally, for a number of patients, several years passed without additional visits after the initial treatment, but later most of them ended up receiving vitreoretinal surgery, which suggests poor compliance causing irregular follow-up in this small patient population [57]. Other studies have noted that younger DM patients have higher probability of not attending DR screening; the average age of our DR patients was between 49 and 63 over the observation period across all treatment modalities, which indicates that the same challenges prevailed among this age group [58].

Ten years ago (2011/2012), low adherence to DM patient screening was reported in Norway. The study indicated that neither the Norwegian General Practitioner Guidelines nor the Liverpool Declaration had an impact on detecting the disease, because of lack of regular eye screening as well as delayed referral [59]. This could also be the case for the current practice and should be further explored. Recent studies on DR patients’ adherence to treatment also showed that age, primary care physician and racial or ethnic background were associated with low adherence to treatment, affirming the risk among this patient group considering the cosmopolitan area the hospital serves [60,61].

With the increasing cost of treating DR patients, it will probably be necessary to re-evaluate the cost-effectiveness of available treatments, considering the possible risks for a lifetime or specified time of treatment/effect. It should also be emphasized that DR is potentially preventable, and appropriate screening programs must be established.

The strengths of our study include the existence of reliable administrative data over a relatively long period (13 years), enabling us to observe time-dependent changes in the pattern of DR treatment. There are also important limitations to the study. First, our administrative data could not capture disease progression or visual outcomes during treatment, and patients receiving multiple treatments were included. Second, it should also be kept in mind that choice of treatment was determined by clinical guidelines specific to our clinic and that different treatment protocols may have brought other results. Third, anti-VEGF injections may be administered to patients before vitreoretinal surgery to decrease the chance of intra-operative complication; such pre-operative injections may have lowered the average IVI frequency in the IVI group. Finally, the ICD-10 coding system does not differentiate between DR and DME.

## 5. Conclusions

Improved visual outcomes that have been demonstrated with IVI have led to transitioning from laser to IVI treatment for DR. We show that the need for IVI therapy has greatly increased hospital utilization and the cost of treating DR, except for a decline in the resource-demanding vitreoretinal surgery. An appropriate IVI drug choice that balances patient needs with economic costs would be a viable strategy in reducing the disease and cost burden in these patients. Nonetheless, an alternative effective regimen of IVI delivery can be a valuable trade-off for outcomes and economic benefit in lessening the frequency burden.

## Figures and Tables

**Table 1:** Unit costs for diabetic retinopathy treatments in Norway.

Item	Unit Cost in EUR (Excluding VAT)	Source
DR treatment per treatment per patient		
Laser	164.25	36R (The Norwegian Directorate of Health (DofH), 2021) [27]
Intravitreal injection	137.45	NoMA Unit cost database
Vitreoretinal Surgery	8504.59	36E (DofH, 2021) [27]
Visit to specialist cost	70.22	NoMA Unit cost database
Intraocular pressure measurement cost	70.25	NoMA Unit cost database
Intravitreal injection drugs per injection per patient		
Bevacizumab	38.24	NoMA Drug cost database
Ranibizumab	338.89	NoMA Drug cost database
Aflibercept	326.75	NoMA Drug cost database
Dexamethasone implant	1075.08	NoMA Drug cost database
Patient time and transport		
Transport cost per journey	58.17	NoMA Unit cost database
Patient time—cost per hour	23.61	NoMA Unit cost database

NoMA—Norwegian Medicines Agency 2020.

**Table 2:** Average number of treatment procedures (standard error) per patient per year and the total number of patients treated per year for diabetic retinopathy.

	2005	2006	2007	2008	2009	2010	2011	2012	2013	2014	2015	2016	2017	2018	Average 2005–2018
Laser	1.09 (0.14)	1.81 (0.17)	1.87 (0.10)	1.88 (0.10)	1.65 (0.10)	1.72 (0.13)	1.04 (0.08)	1.24 (0.09)	1.04 (0.10)	1.38 (0.11)	1.07 (0.08)	0.95 (0.07)	0.64 (0.05)	0.54 (0.05)	2.2 (0.04)
Intravitreal injections	0.08 (0.03)	0.02 (0.01)	0.10 (0.02)	0.16 (0.03)	0.35 (0.06)	0.70 (0.10)	1.95 (0.18)	2.05 (0.15)	2.74 (0.20)	2.78 (0.17)	3.20 (0.16)	4.33 (0.17)	4.36 (0.15)	4.73 (0.14)	4.7 (0.07)
Vitreoretinal surgery	0.54 (0.06)	0.40 (0.05)	0.20 (0.03)	0.22 (0.03)	0.20 (0.03)	0.22 (0.03)	0.27 (0.04)	0.16 (0.02)	0.18 (0.03)	0.17 (0.03)	0.11 (0.02)	0.08 (0.01)	0.07 (0.01)	0.05 (0.01)	1.2 (0.02)
Treated patients	91	128	260	265	217	208	249	300	315	318	405	487	580	611	

**Table 3:** Average costs per patient per year in each treatment group.

Year	Treatment	Observed Average (EUR)	Bootstrapped Standard Error	95% Confidence Interval	Total Cost per Patient
2010	Laser	899	59	785–1014	2906
	Intravitreal injection	1116	115	892–1341	
	Vitreoretinal Surgery	10,348	555	9261–11,436	
2011	Laser	750	43	666–833	3737
	Intravitreal injection	1977	141	1701–2253	
	Vitreoretinal surgery	10,882	733	9445–12,318	
2012	Laser	844	50	747–941	2935
	Intravitreal injection	1752	94	1567–1937	
	Vitreoretinal surgery	9308	325	8672–9945	
2013	Laser	862	57	751–973	3592
	Intravitreal injection	2604	146	2316–2891	
	Vitreoretinal surgery	10,226	554	9140–11,312	
2014	Laser	1043	61	924–1161	3831
	Intravitreal injection	2774	131	2517–3032	
	Vitreoretinal surgery	12,188	1168	9898–14,478	
2015	Laser	971	52	868–1073	3518
	Intravitreal injection	2960	115	2735–3184	
	Vitreoretinal surgery	10,438	678	9109–11,766	
2016	Laser	1073	52	972–1174	3928
	Intravitreal injection	3645	115	3419–3871	
	Vitreoretinal surgery	9737	607	8547–10,927	
2017	Laser	880	45	792–967	3664
	Intravitreal injection	3437	103	3236–3638	
	Vitreoretinal surgery	10,377	611	9180–11,573	
2018	Laser	836	41	755–917	3665
	Intravitreal injection	3442	89	3268–3616	
	Vitreoretinal surgery	10,357	670	9043–11,670	

All values shown are rounded to the nearest Euro.

## Data Availability

Not applicable.
